# A perspective on the regulation of cultivated meat in the European Union

**DOI:** 10.1038/s41538-025-00384-0

**Published:** 2025-02-08

**Authors:** Alessandro Monaco

**Affiliations:** Chair of Food Law, Faculty of Life Sciences: Food, Nutrition and Health, University of Bayreuth, Fritz-Hornschuch-Straße 13, 95326 Kulmbach, Germany

**Keywords:** Law, Science, technology and society

## Abstract

In the European Union, the adequacy of the novel food framework to deal with cultivated meat has been questioned by several political initiatives at member state and Union levels. This contribution argues that the novel food framework is fit for regulating the entrance into the market of cultivated meat and that the use of the precautionary principle to ban cultivated meat production and commercialization is not justified. It then illustrates how existing regulatory provisions already provide the EU gastronomic heritage with adequate safeguarding, if and when cultivated meat will enter the EU market.

## Introduction

The production of cultivated meat by growing animal cells for human consumption under lab conditions has the potential to revolutionize the food sector^1^. In the European Union (EU) cultivated meat is legally classified as a novel food. Novel foods are foods not used for human consumption to a significant degree within the Union before 15th May 1997 that can be classified in one of the ten novel food categories^2^.

Novel foods require pre-market approval before being placed on the market. Once a novel food is included in the Union List of Novel Foods it can be legally sold across the Union. An authorized novel food reaching the market is as safe as a non-novel food. However, with respect to cultivated meat, concerns have been raised within the EU about the effectiveness of the novel food framework in properly regulating its disruptive potential^3^. Consequently, political initiatives have emerged against cultivated meat.

In the European Protein Strategy, adopted in October 2023, the European Parliament stated that the novel food framework is not fit for evaluating the ethical, social, environmental and economic challenges posed by cultivated meat^4^. On December 16th 2023, a law banning cultivated meat production and commercialization entered into force in Italy^5^. Based on the precautionary principle, the law was adopted to protect human health and the country´s gastronomic heritage. On January 22nd, 2024, the delegations of Italy, Austria, France, and eleven other EU member states issued a note to the Council of the EU, expressing concerns over the safety of cultivated meat^6^. The note advocates for a more comprehensive approach to cultivated meat, addressing ethical, economic, and social considerations. It invites reflection on potential implications for public health and sustainability, questioning the adequacy of the existing legal framework in addressing these concerns and calling for the application of the precautionary principle.

This Perspective contributes to the ongoing discussion over cultivated meat regulation in the EU by briefly addressing two topics: the suitability of the novel food framework to deal with cultivated meat and the ability of existing regulations to safeguard traditional high-quality foods in case of widespread adoption of cultivated meat.

## Considerations on the regulation of cultivated meat

### Suitability of the EU novel food framework to assess cultivated meat safety

The novel foods authorization process aims to verify that novel foods do not pose safety risks to human health based on available scientific evidence. It ensures that the intended use of the novel food does not mislead consumers, especially in cases where the novel food is meant to replace another food and undergoes a significant change in nutritional value. Also, novel foods should not differ in a way that their consumption would be nutritionally disadvantageous for the consumers^7^.

The authorization procedure consists of a risk assessment conducted by the European Food Safety Authority (EFSA), which produces a Scientific Opinion, and of a risk management phase, where representatives of the member states vote on the final authorization of the novel food in the Standing Committee on Plants, Animals, Food and Feed (PAFF). This vote is based on a draft authorization prepared by the Commission taking into consideration the Scientific Opinion, the precautionary principle, and other legitimate factors. Throughout the procedure, transparency is ensured by making the EFSA´s Scientific Opinion and the conditions of each authorization publicly available. The novel foods authorization procedure is supposed to last eighteen months, but it can extend up to three years as EFSA has the discretion to stop the procedure to require more information if needed^8^. The scope of the authorization is accurately defined and concerns individual products manufactured under specific conditions.

The Italian law mentioned above, as well as critics of cultivated meat in general, make direct reference to the precautionary principle, arguing that cultivated meat might pose unknown risks to consumer health. According to the Italian government, a ban on cultivated meat production and commercialization is justified because there is little research considering the risks derived from its consumption. The lack of research would constitute reasonable grounds for the use of the precautionary principle. However, the alleged absence of research on cultivated meat consumption is not enough to justify the application of the precautionary principle^9^.

In EU food law, the precautionary principle is defined in Article 7 of Regulation (EC) No. 178/2002^10^ and states that under specific circumstances, when, following an assessment of available scientific evidence, uncertainty over the possibility of harmful effects persists, temporary risk management measures can be adopted to protect human health. In short, it requires a scientific assessment to be conducted on available evidence before temporary measures are adopted.

“Cultivated meat” is a collective term referring to cell-based products that differ in terms of cell origin, growth media, and uses. So far, only some products of cell culture from plant cells have been authorized in the EU. As of today in the EU, only one application concerning a cultivated meat product has been submitted by the company Gourmey in 2024^11^. The assessment of the application is still in its early phases^12^. The Gourmey´s application (or other upcoming applications) will make it possible to conduct an assessment of the available scientific evidence. A cultivated meat application presenting inconclusive safety data would be negatively evaluated by EFSA without even an appeal to the precautionary principle. In contrast, other submissions could meet the safety requirements and be assessed positively. Every submission is considered individually, and the scope of the authorization is clearly defined. For example, for the novel food “apple fruit biomass from cell culture”, only one apple variety is allowed; other apples might require a separate assessment^13^. Since the risk management measures must be provisional and proportionate, the precautionary principle cannot justify an outright ban on all cultivated meat products. At worst, a temporary ban could be issued on a specific cultivated meat product if scientific evidence demonstrated a risk to human health after its placement on the market following a novel food authorization.

Last but not least, political representatives have the right to vote on the final authorization for a novel food. As of today, if the representatives of the fourteen countries that endorsed the Note to the Council voted against the authorization in the PAFF Committee, a cultivated meat application could be rejected or not approved even after a positive Scientific Opinion from EFSA^14^. A trajectory observed in the EU with genetically modified organisms^15^.

### Ability of existing regulations to safeguard traditional high-quality food products

Opponents of cultivated meat argue that the novel food framework is not suitable to assess the (potentially disruptive) socio-economic impact of cultivated meat. In particular, the cultural heritage associated with traditional, high-quality foods would be threatened by the entrance into the market of cultivated meat. These concerns are used together with the precautionary principle to justify the adoption of bans, as in the case of the Italian law^3^. Unsurprisingly, these politics are always backed by farmers and sector organizations such as the Italian Coldiretti^16^, or the Confédération Paysanne in France^17^. However, an outright ban on cultivated meat cannot be sustained on the grounds of protecting the European gastronomic heritage either. Beyond the scope of the novel food framework, which primarily ensures the safety of novel foods, other regulatory mechanisms at the EU level already provide a structure to safeguard local gastronomic heritage and high-quality traditional foods.

Products like Prosciutto di Parma DOP, Jambon de Bayonne DOP or Vaca de Extremadura PGI are regulated under the Geographical Indications (GIs) framework. GIs are forms of protection of intellectual property rights for food products whose qualities are linked to the area of production and to a particular savoir-faire. GIs aim to defend the brands associated with traditional, high-quality products whose characteristics are defined by a consortium of producers within the product specifications. It is quite unlikely that cultivated meat will ever be allowed to be used in a GI. Ultimately, however, producers decide whether the product specifications should be changed, giving them the power of choice^18^.

Additionally, similarly to what happens with genetically modified plants, cultivated meat products will hardly be considered compatible with the organic framework, which aims to offer consumers foods produced using “*natural substances and processes*”^19^. In Annex II Part II of Regulation (EU) 848/2018 on organic production, reproduction of organic livestock is required to only use natural methods, avoiding forms of artificial reproduction such as cloning or embryo transfer^20^. Techniques of cell culture would not be considered differently. The organic certification will thus remain a prerogative of meat produced from traditional livestock.

Looking at the future, two, non-mutually exclusive scenarios appear plausible for cultivated meat. First, cultivated meat might compete with conventionally produced, low-priced meat. Assuming it will always be labelled adequately, since the labelling requirements will be specified in the novel food authorizations, the competition between these products will hinge on consumer perception and acceptance. Alternatively, cultivated meat could occupy a niche market of unusual products, such as the cultivated quail meat currently under regulatory revision in Australia^21^. In both scenarios, cultivated meat is unlikely to replace the traditional high-quality products that are integral to European gastronomic heritage.

## Conclusions

The novel foods framework provides a robust and rigorous approval process to assess potential risks for human health. It separates the scientific evaluation of available data from the final, political decisions, and it is designed specifically to deal with innovative products like cultivated meat. In January 2024, European Commissioner Kyriakides again stated her confidence that the novel food framework is suitable for assessing the safety of cultivated meat and is able to address the concerns raised by EU member states^22^. This confidence is strengthened by the new dedicated guidelines for the assessment of products of cellular agriculture, which were published in September 2024^23^, after more than one year of consultations with stakeholders^24^. There is no reason to invoke the pre-emptive application of the precautionary principle, especially considering that no cultivated meat product has been fully assessed under the novel food framework yet.

If the debate over the safety of cultivated meat is premature, the conflict between cultivated meat and traditional food products is arguably artificial, due to the mechanisms of protection that traditional foods already enjoy in the EU. Labelling of cultivated meat will always be transparent for consumers, as their legal names and conditions of use will be specified in the novel food authorizations. Regulatory instruments such as GIs and the organic framework will safeguard high-quality products and the desire of consumers for foods produced “naturally”. Lastly, pre-emptive bans adopted by EU member states, such as Italy, and similar legislative efforts in France, Romania, and Poland, are likely incompatible with internal market rules^25^.
